# People who use drugs in rehabilitation, from chaos to discipline: Advantages and pitfalls: A qualitative study

**DOI:** 10.1371/journal.pone.0245346

**Published:** 2021-02-05

**Authors:** Nadine Mahboub, Gladys Honein-AbouHaidar, Rana Rizk, Nanne de Vries

**Affiliations:** 1 Department of Nutrition and Food Sciences, Lebanese International University, Beirut, Lebanon; 2 Department of Health Promotion, Maastricht University, Maastricht, The Netherlands; 3 Hariri School of Nursing, American University of Beirut, Beirut, Lebanon; 4 Refugee Health Program, Global Health Institute, American University of Beirut, Beirut, Lebanon; 5 Institut National de Santé Publique, d’Epidémiologie Clinique, et de Toxicologie (INSPECT-LB), Beirut, Lebanon; 6 Department of Health Promotion, Maastricht University, Maastricht, The Netherlands; University of Arkansas for Medical Sciences, UNITED STATES

## Abstract

Evidence-based models emphasizing on lifestyle behaviours for the treatment of drug use is still in its infancy. The development of multicomponent effective drug use intervention programs as part of health promotion is crucial to decrease risk of relapse. This study aims at exploring the lifestyle practices including dietary intake, physical activity and sleep of people who use drugs undergoing residential rehabilitation treatment in Lebanon with its perceived benefits and pitfalls. A purposive sample of 18 males and 9 females at different stages of recovery from drug use in rehabilitation centers participated in the qualitative discussions. The six phases thematic analysis revealed three themes: chaotic lifestyle, structuredlifestyle, benefits and pitfalls, and suggestions for making rehabilitation a better experience. Participants discussed their chaotic lifestyle during addiction with poor food intake, disrupted sleep and low physical activity moving to a more disciplined routine enforcing normality in lifestyle practices with social and professionlprofessional support. The early phases of treatment were marked with increased food intake and weight gain perceived as a health indicator and the sole divergent from drugs, moving towards more structured meals and efforts to lose weight in later stages. Lack of variety of Physical activity programs taking into consideration the motivational differences among the participants was also highlighted. Measures for improving rehabilitation services in terms of promoting healthy eating behaviours and environmental control were thoroughly addressed. These findings shed the light on the challenges faced in maintaining a healthy lifestyle in rehabilitation centers and the necessities of addressing them to improve the overall rehabilitation experience, prevent relapse and inform the development of future targeted intervention programs tackling all aspects of behavioural changes.

## Introduction

Illicit drug use among young people is epidemic and remains a significant public health concern. It poses harmful consequences on the individual’s emotional, social and psychological status leading to higher risks for diseases and mental disorders [1]. In addition to this, it is associated with unhealthy lifestyle practices including poor dietary behaviours, changes in physical activity and sleep problems leading to substantial health problems and poor quality of life.

Drug use greatly affects individual’s dietary behaviour, and compromises their nutritional status [2]. In general, this population has a disrupted and chaotic lifestyle, where money is usually spent on drugs rather than food [2]. Brain changes that occur due to psychological and neurobiological factors in substance use disorder, make cravings for the substance more important than anything else in the person’s life [3]. This severely affects the food intake of People Who Use Drugs (PWUD)’ where most of them have a low nutrient-dense food intake with sweet cravings [4–6] and decreased body mass index (BMI) [7–10] putting them in a mild to moderate malnutrition status [11].

Physical activity is another lifestyle practice affected by drug use. The scarce evidence on PWUDs’ involvement in physical activity is controversial. Some suggest low participation in structured sports [12, 13], while others show active participation in an attempt to stay healthy and divert from the use of drugs as a self-care strategy [14–16].

Sleep is negatively affected by drug use [17–20]. An estimated 10–15% of people with chronic sleep disturbance have underlying substance use disorders, and this is affected by the type of drug used [20–22].

Once referred to inpatient rehabilitation services, i.e. detoxification followed by psychotherapy and behavioural modification therapies [23], addressing lifestyle practices and improving the quality of life of PWUD are crucial to decrease the risk of relapse [24].

During rehabilitation, dietary behaviours and nutritional status fluctuate depending on the stage of recovery [25]. In the early stages, binging on sugars is observed as a replacement for the drugs compared to more structured eating behaviours later [26–28].

Physical activity is another potential non-pharmacological treatment for substance use disorder [29]. It was shown to reduce sufferings from withdrawals, anxiety, and depression, in addition to improving self-confidence with a sense of the new quality of life [24, 30, 31]. Nevertheless, the controversy around benefits of physical activity during rehabilitation remains in light of the scarcity of studies exploring this issue.

The sleep of people who use drugs undergoing treatment, especially in rehabilitation services, has also received little attention. The dearth of studies are conducted on persons with opiate addiction receiving methadone treatment, showing inadequate sleep quality and quantity which could arise from a mix of contributory causes like psychopathological problems, nicotine use, duration of opiate use, in addition to methadone itself that produces sleep abnormalities [21, 32, 33]. Furthermore, attention to proper nutrition could be a key to better sleep during rehabilitation. Studies have shown that specific macronutrients might influence the person’s sleep quality. Indeed, there is a negative association between the consumption of foods high in refined carbohydrates, a common practice among people who use drugs in rehabilitation, and sleep duration [34, 35].

Research on best practices for disseminating evidence-based substance use disorder (SUD) treatments into practice is still emerging [36]. Models used for recovery from addiction mainly emphasized on basic pharmaco and psycho-social elements like understanding and accepting self, family and peer support and community envolvement [37], with little emphasis on lifestyle behaviours tailored to meet the individuals needs and preferences. Emerging evidence continues to shed light on the benefits conferred by physical activity, balanced nutrition and sleep on mental health and wellness, so alternative treatment interventions incorporating these three aspects fashions a comprehensive approach to mental health treatment with numerous benefits [38]. The need for the development of multicomponent effective drug use intervention programs as part of health promotion is crucial to decrease risk of relapse [37].

The present study was conducted in Lebanon, a small high-middle income country in the Eastern Mediterranean region. Despite the various predisposing factors for drug use including internal and regional armed conflicts for over three decades, studies on drug use patterns in Lebanon are scarce [39–41]. To our knowledge, no previous studies were conducted on the in-patient rehabilitation services offered in the country.

This study aims at exploring the lifestyle practices including dietary intake, physical activity and sleep of PWUD undergoing treatment (detoxification followed by in-patient rehabilitation) in Lebanon and perceived benefits and pitfalls. Ultimately, the findings will inform the development of future targeted intervention programs.

## Materials and methods

### Design and approach

This is a descriptive qualitative study based on focus group discussions (FGDs) with a sample of PWUD undergoing treatment in rehabilitation centers in Lebanon. Ethical approval was obtained from the American University of Beirut (SBS-2018-0424) and the Lebanese International University (LIUIRB-180122-NB1) Institutional Review Boards. A written consent was handed and signed by the participants. This consent form explained the purpose of the study, the process of data collection, and that the participants can withdraw from the study at any time. Also, their consent for audiorecording the sessions and quoting them in the final manuscript was sought.

### Sample

A purposive sampling approach was used to recruit participants from drug rehabilitation centers.The criteria of selection were adult from both genders (above 18 years of age), Lebanese and active people who use drugs seeking treatment. We excluded non-Lebanese participants, as they may have different triggers for addiction and less optimal rehabilitation conditions (e.g. immigrants lacking family support essential for treatment), and participants below the age of 18. Seven FGDs were conducted on participants in rehabilitation centers as they were acquainted with each other and shared a similar experience and setting.

### Recruitment and data collection

We approached the seven operating rehabilitation centers in Lebanon. Only three out of seven granted us approval to collect data from their patients. From each center, eligible participants who consented to participate in the discussions were approached and informed about the objectives, the methods of the study,and about their right to withdraw at any time. Two groups of participants were chosen: those who have been in rehabilitation for less than six months (early recovery) and those who completed more than six months (late recovery). The early recovery groups ranged from four to five participants, while the late recovery ones ranged from three to six participants. Two of the centers provide services for males solely and one for females. In total, twenty- nine persons met the eligibility criteria. One person refused to participate, one left during the discussion, and twenty- seven finished the FGDs.

The two researchers NM and GHA introduced themselves to the participants and handed them the written consent. No compensation was offered to the participants for their time spent in the FGDs. A semi-structured discussion guide containing open questions was used; however, probing questions arose to delve into important points raised by the participants (S1 Appendix). All discussions were conducted in Arabic and in a private room in the center. One researcher was taking notes and the other was moderating. The discussion was started by asking each participant to introduce him/herself and since participants were acquainted with each other, they discussed their lives freely during addiction and the factors leading them to seek treatment. Followed by this, the moderator guided the discussion reaching the daily routines in the center including assigned chores, their food intake and choices, factors affecting their intake, weight change experiences, sleep and physical activities. Finally, the need for nutrition education in the center and the best way to deliver it was also addressed. All FGDs were conducted before the analysis. Following each FGD, NM and GHA had a debriefing session to discuss the yield. Saturation was reached after seven FGD.

### Data analysis

Sociodemographic characteristics were analyzed using SPSS, version 21 Software. All FGDs were transcribed by NM and EM. We used the six phases thematic analysis approach recommended by Braun et al [42] (S2 Appendix). Themes and sub-themes were identified by NM and GHA without the use of a qualitative data analysis software, and corresponding quotes were translated and saved in a data repository. Representative quotes were cited in the manuscript. This study followed the consolidated criteria for reporting qualitative studies (COREQ) (S3 Appendix).

### Increasing rigor

We ensured that credibility and reflexivity were observed. In terms of credibility, focus group moderators shared the same first language for ease of communication. All conversations were audio recorded, transcribed verbatim, translated into English, and used as the main data repository. We invited all the centers that exist in Lebanon, but we conducted 7 FGDs representing all the centers that consented to participate. More importantly, we believe we reached saturation given that no further findings emerged in the final FGD. In terms of reflexivity, to avoid any undue influence, the moderators (GHA and NM) had no prior relationship with participants. Further, during focus group moderation, the roles of the moderator and observer were disclosed to participants. Finally, all team members were involved in the analysis so as to avoid bias interpretation of the results.

## Results

### Sociodemographic characteristics of study participants

In total seven FGDs were conducted. Eighteen males and nine females in recovery from substance use disorder in three different rehabilitation centers in Lebanon participated in this study. Demographic information of the sample are detailed in Table 1. Fifty-two percent of the participants fell in the early recovery stage (less than six months).The mean age of the sample was 30.1±6.5 years, with the majority (52%) having a university-level education. All participants were unemployed during rehabilitation, with 74% being single at the time of the assessment. On average, the participants initiated drug use at the age of 19.48± 18.91, and used drugs for 12.39±7.71 years. The vast majority of the sample (89%) used drugs more than 3 times daily, half of them (52%) injected drugs inaddition to other use methods simultaneously. Cocaine was the most frequent drug used, followed by heroin, tranquillizers, and cannabis. Other substances used included pain killers, hallucinogenics, and stimulants.

**Table 1:** Demographic information of participants of the Focus Group Discussions (FGD).

		Center 1 (n = 9)	Center 2 (n = 8)	Center 3 (n = 10)	Total (n = 27)
**Age (mean ±SD)**		29.2±6.1	28.6±5.4	32.1±7.7	30.1±6.5
**Gender (%)**	Male	0 (0)	8 (100)	10 (100)	18 (66.7)
	Female	9 (100)	0 (0)	0 (0)	9 (33.3)
**Treatment duration (%)**	Early recovery (<6 months)	5 (55.6)	5 (62.5)	4 (40.0)	14 (51.9)
	Late recovery (>6 months)	4 (44.4)	3 (37.5)	6 (60.0)	13 (48.1)
**Educational level (%)**	Elementary/ intermediate	0 (0)	6 (75.0)	4 (40.0)	10 (37.0)
	Secondary	1 (11.1)	0	2 (20.0)	3 (11.1)
	University	8 (88.9)	2 (25.0)	4 (40.0)	14 (51.9)
**Employment(%) (pre-rehabilitation)**	Unemployed/ Retired	3 (33.3)	0	2 (20.0)	5 (18.5)
	Employed	3 (33.3)	6 (75.0)	3 (30.0)	12 (44.4)
	Self-employed	1 (11.1)	2 (25.0)	5 (50.0)	8 (29.6)
	Student	1 (11.1)	0	0	1 (3.7)
	No response	1 (11.1)	0	0	1 (3.7)
**Marital status (%)**	Single	5 (55.6)	8 (100)	7 (70.0)	20 (74.1)
	Married	1 (11.1)	0	0	1 (3.7)
	Divorced/Separated	3 (33.3)	0	3 (30.0)	6 (22.2)
**Type of drug use (%)**	Drug use only	5 (55.6)	2 (25.0)	4 (40.0)	11 (40.7)
	Drug injection only	1 (11.1)	0	1 (10.0)	2 (7.4)
	Drug use and injection	3 (33.3)	6 (75.0)	5 (50.0)	14 (51.9)
**Substances used pre-treatment (%)**	Heroin	4 (44.4)	6 (75.0)	8 (80.0)	18 (66.7)
	Cocaine	7 (77.8)	7 (87.5)	8 (80.0)	22 (81.5)
	Crack	6 (66.7)	7 (87.5)	8 (80.0)	21 (77.8)
	Buprenorphine (Tidigesic)	3 (33.3)	5 (62.5)	6 (60.0)	14 (51.9)
	Dextropropoxyphene	1 (11.1)	2 (25.0)	0	3 (11.1)
	Amphetamines	5 (55.6)	5 (62.5)	4 (40.0)	14 (51.9)
	Tranquilizers	5 (55.6)	6 (75.0)	7 (70.0)	18 (66.7)
	Barbiturates	1 (11.1)	3 (37.5)	3 (30.0)	7 (25.9)
	Cannabis	6 (66.7)	6 (75.0)	7 (70.0)	19 (70.4)
	Other	6 (66.7)	5 (62.5)	6 (60.0)	17 (62.9)
**Frequency of drug use (%)**	More than 3 times daily	9 (100.0)	8 (100.0)	7 (70.0)	24 (88.9)
	One to 3 times daily	0	0	3 (30.0)	3 (11.1)
**Age at first use (mean±SD)**		29.00±31.69	14.87±1.45	14.60±1.64	19.48±18.91
**Duration of drug use (mean±SD)**		9.38±5.87	11.00±6.66	16.33±8.93	12.39±7.71

### Rehabilitation centers’ characteristics

The three rehabilitation centers were mainly governed and subsidized by the Ministry of Social Affairs in Lebanon, except for the medications that were under the jurisdiction of the Ministry of Public Health. The centers shared common criteria for accepting patients including completing a detoxification program prior to admission, willingly accepting to go through rehabilitation, and being free of any contagious disease such as tuberculosis. A urine test is required to confirm detoxification, and is done prior to admission. Once admitted, patients needed to follow strict discipline in terms of sleeping hours, meal time, occupational tasks, and restricted access to television and social media. Further, they were not allowed to go out for three months, afterwhich they were allowed occasional supervised visits to their families. Following each visit, a urine test is done to rule out the use of drugs. At the women’s center, children were not allowed to stay with their mothers. They only see them during visitation, which is allowed after three months of admission.

The care providers are social workers, psychologists and psychiatrists offering evidence- based services such as social programs, cognitive behaviour therapy, and pharmacotherapy. At one center, patients received Christo therapy which is a faith-based intervention focusing on prayers as a mean to overcome craving and addiction.

### Emerging themes

The yield of the discussions can be summarized into the following three themes: chaotic lifestyle: reasons and consequences of drug use, structured lifestyle, benefits and pitfalls, and suggestions for making rehabilitation a better experience. Within each theme, we identified several sub-themes and we noted when they differed by gender and recovery stage.

### Chaotic lifestyle: Reasons and consequences of drug use

#### Sub-themes

1- “We’re not used to tackling life without drugs.”

For most participants, drugs were the way out from low self-esteem, depression and stressful experiences in life, such as family conflicts. Drug use was the gateway for fitting in with friends and family members. There were few exceptions particularly two females who indicated that weight gain was the trigger for drug use, while two others started as a result of chronic pain and usage of pain killers.

“*I started my drug use in my teenage*, *I wanted to keep pace with our generation*, *and that’s how it went*.*” (0410; female 26 years)*

“*After I got out of here*, *I relapsed again because of my weight gain*.” *(0407; female 36 years)*

2- Chaotic lifestyle “No discipline, no discipline, no time, nothing, nothing.”

Eating habits were messed up. Most indicated rarely eating and were craving mainly for carbohydrates. All preferred to spend the money on drugs rather than food. Sleeping patterns were also chaotic, where drugs had deleterious consequences leading to disrupted sleep. Consequently, many had major health complications.

“*Mostly*, *its sugar*, *bonbons and juices” (0410; female 26 years)*.

Only two participants practiced physical activity during their substance use disorder period. One male had a passion and interest for competitive sports but despite this, participation levels tended to cutail dramatically once heavy drug consumption set in. On the other hand one female reported that she had routinely participated in physical activity despite her substance use disorder, which in turn played a role in preventing extreme weight loss as a result of the drugs.

“*I took a personal trainer and nutrition course before I started my addiction*. *I used to go to competitions in sports*. *When I started drugs my weight changed from 88 to 55Kg*. *I was depressed*, *stayed at home all times and stopped going to the gym*.*”(0207; male 19 years)*

3- Reasons and motivations for rehabilitation “Got sick of the life with drugs.”

Most participants had a tipping point leading to rehabilitation, for some extrinsic, for others intrinsic factors. Three participants stated being coerced by legal authorities to seek treatment and fear of imprisonment led them to rehabilitation. Five others indicated that family and peers motivated them to seek treatment.

“*At first*, *I had to be admitted to a rehab because of the drug court*.*” (0409; female 26 years)*

Fear was the internal driver to seek treatment for three participants. Mainly it was the fear of losing a job or losing a life due to overdose. Many indicated being fed-up with the chaotic lifestyle that led them to rehabilitation. For others, remorse after relying on violence or theft to acquire the drug triggered this desire to seek help for drug cessation.

“*I got fed up and tired of the life of drugs; I wanted to save myself*.*” (0302; male 24 years)*

Five participants indicated that because they had a relapse from previous rehabilitation experience, they were challenged to pursue further treatment.

Females specifically noted that treatment was the only way not to lose the custody of a child. Three of the participants were mothers; two had already lost the custody of a child because of drug addiction, while the third one was pregnant during addiction. All wanted to regain or resume their roles as mothers after treatment.

#### Structured lifestyle, benefits and pitfalls

The shift from chaotic to structured lifestyle was the hallmark for this period.

In rehabilitation, participants became more connected to daily life routines, had more social and professional support. This period was characterized as mostly very welcomed albeit its pitfalls.

### Sub-themes

1- Eat, exercise and sleep.“Our daily routine.”

All participants reported living a more structured lifestyle. The mornings started by waking up early and meeting for breakfast followed by completing their assigned chores like cleaning, cooking, or gardening. After lunch, they did recreational tasks like arts especially for females, psychotherapy meetings, followed by dinner and early sleep.

During the early stages of recovery (1–6 months), this lifestyle helped them gain weight. This was perceived as an indicator of health replacing what they lost during addiction; and this in turn increased their self-confidence and self-image

“*I gained 16 Kg in two months*, *huge number*.*” (0410; female 26 years)*

“*I’m actually satisfied that I’m putting on some weight*.*” (0304; male 23 years)*

Eat

Meals were part of the disciplined lifestyle as participants described having three communal meals at a fixed time. Most participants in the early stages of rehabilitation (1–6 months) expressed that they ate large amounts of food during meals and craved for sweet and junk foods that were used as a replacement for drugs.

“*Outside rehab I used to eat a sandwich; here I have my full breakfast*. *At lunch*, *instead of eating a plate*, *I eat two or three*. *Some people around here binge eat as a way to overcome the stress and need for drugs*.*” (0401; female 23 years)*

Some blamed their binging on food on their frustration from the strict environment in the rehabilitation centers; they ate out of boredom. Eating became the only source of diversion from drugs. Four participants stated that the menu offered in the center was healthy and constituted of vegetables, grains and some proteins when available. Three others perceived the high amount of carbohydrates given and little protein with no limited portions as unhealthy.

“*The food here is way better than food outside*, *it’s healthier*.*” (0209; male 21 years)*

“***The available*** *foods are not so healthy*.*” (0405; female 25 years)*

Females showed frustration regarding the weight gain, and to some this was a reason for relapse.

In the late recovery stage (6–12 months), meal structuring became a part of their daily routine and food was no more seen as a substitute for drugs where most participants reported some struggle in terms of food intake control. Consciousness of the weight gain and desire to lose the extra weight was frequently expressed in this stage of the treatment. Weight gain and increased food intake was no more seen as a health indicator, rather as a cause for some for drug relapse leading to frustration and self-hatred.

“*I see people suffering from their extra weight*. *If I ever put that weight on*, *I would do drugs for a month to lose that extra weight and then I’ll quit*.*” (0212; male 37 years)*

Exercise

Physical activity is a mandatory daily or weekly routine in all centers. Participants had a positive attitude towards physical activity and indicated marked physical, psychological and craving benefits from it. On the other hand, some reported that it was a daily routine that was not enjoyed and boring.

“*It is a repetition*, *every week it is the same*, *it is a routine*: *running*, *stretching*.*” (0407; female 36 years)*

Sleep

Upon admission, all participants suffered from poor sleep. In the rehabilitation, their sleeping patterns were more disciplined in terms of timing and duration. Excessive sleeping hours especially during the day was reported to the caregivers as it was perceived as a sign of improper coping and possible relapse to drugs. Some females expressed the need for more sleeping hours.

2-Therapy

In terms of therapy, in the religious centers, prayers (Christo-therapy) played a major part of the treatment. Psychotherapy was also included in the daily routine, where weak points of the participants leading to relapse were identified and worked upon.

3-Support system

Most participants pointed to three types of support: social environment, professional and peer. This support was very important in helping participants hold back their cravings for food and drugs.

Creating a supportive social environment included close monitoring of potential triggers to drug use. This type of support embeded security and safety within participants for sustainability and drug relapse prevention.

Most of the participants expressed their gratitude to the excessive professional support and care offered by the care providers through active listening, empathy and lack of judgement and hostility.

Peer support was also cited as a positive supporting system to cope with mainly craving. The pre-set knowledge of the obstacles that will be faced in the different stages of the treatment insured better coping within the participants in addition to them supporting other peers in their first experience. This applied largely to the increased food intake, sweet cravings and weight increase that was faced in the early stages of the treatment, in addition to drug craving.

### Suggestions for making rehabilitation a better experience

Whilst residential rehabilitation treatment centers provide a stable environment to target multiple health risk behaviours, these services tend to focus mainly on the drug and alcohol use disorders of the individuals. Other factors like smoking, healthy eating and exercise should be addressed and tackled as part of the daily routine. The participants expressed the need to tailor a program addressing three needs:

1-Nutrition

Two types of suggestions emerged. One in relation to environmental control and the second was promoting healthy eating behaviours. Participants expressed the need to have “*healthy snacks”* available at all times as a means to decrease the sweet cravings and limit the food intake to healthy choices. Others expressed that the rehabilitation centers should have “*dietitians setting healthy daily menus with emphasis on portion control”* as a means to control the weight gain experienced. However, strict discipline was not recommended in the first stages as they have enough rules and having more may offset individuals.

“*A nutrition intervention program”* in treatment centers that provides general nutrition education to all participants in the early stages of the treatment (0–6 months) was desired. An important aspect of this program is to raise awareness about the increase in food intake and weight that the participants might face during the treatment.

In later stages (6–12 months), having an “*individualized consultation”* to members in need of weight monitoring or loss was expressed.

“*Start with general information about nutrition and then it becomes individualized*.” *(0303; male 36 years)*

2- *Physical Activity*

Participants expressed the need for more varied physical activity programs to be administered in the afternoons rather than mornings because this was when craving for drugs was intensified

“*In the morning you wake up calm*, *but in afternoon you have cravings so sports are very important*.*”(0401; female 23 years)*

3-Transitional programs post-rehabilitation

Some participants emphasized on the need to have transitional programs post-rehabilitation where coaching sessions are delivered to prevent relapse.

“*Every drug user needs follow up*. *Every person who leaves the center today will relapse if not now maybe after a year*. *We need to stay protected*. *The one who wants to stop drug abuse has to stay protected all his life*. *He needs follow up all his life*. *“(0207; male 19 years)*.

“Table 2” summarizes more specific quotes related to themes and their subthemes.

**Table 2:** Specific quotes corresponding to the themes and their sub-themes.

Theme	Subtheme	Quotes
***Chaotic lifestyle***	*“We’re not used to tackling life without drugs”*	*“My addiction started because of my parents’ problems and divorce*.*” (0406; female 40 years)*
		*“I wanted to have friends older than me and got used to drugs*.*” (0306; male 24 years)*
		*“Everyday depression*.*” (0201; male 42 years)*
		*“My siblings do drugs too*.*” (0407; female 36 years)*
		*“At first*, *I had gallbladder disease and because of medications I started drugs*.*” (0407; female 36 years)*
	*Chaotic lifestyle “No discipline*, *no discipline*, *no time*, *nothing*, *nothing*.*”*	*“Drugs suppress[ed] appetite” (0308; male 28 years)*
		*“I became anorexic*, *I now weigh 37 kg and I have no power left in me*.*” (0410; female 26 years)*.
		*I was depressed*, *stayed at home all times and stopped going to the gym*.*” (0207; male 19 years)*
		*“I used to go to a dietitian before and during my addiction*. *I kept doing sports during my addiction that is why my weight did not change*.*” (0405; female 25 years)*
	*Coerced “Got sick of the life with drugs*.*”*	*“Got out of prison*, *stayed home then back to prison again*.*” (0307; male 32 years)*
		*“I travelled to Africa and ran out of drugs*, *so I went back to Lebanon and I was advised by my uncle to go to a rehab center so I can get my life together*.*” (0406; female 40 years)*
		*“We got sick of the life with drugs*, *it was painful*, *and we went through a lot*, *we have tried many ways to overcome drug addiction and there was no other solution*. *Most of us here are well educated and well aware that addiction is a disease of no cure but rehab*.*” (0203; male 38 years)*
		*“I don’t steal; how did I do that to my parents*? *What affected me the most is the way I talked with my father*, *I snapped and told him*: *I will break the fridge and the TV*, *just give me the money*.*” (0306; male 24 years)*
		*“Many times*, *I have packed my bag to be admitted to a rehab and then I would leave and go back home*. *I’ve let my parents down a lot*.*” (0307; male 32 years)*
***Structured lifestyle*, *benefits and pitfalls***	*Eat*, *exercise and sleep*. *“Our daily routine*.*”*	*“We wake up in the morning*, *pray*, *have breakfast*, *do chores assigned to us*, *attend a meeting*, *take a break*, *sometimes there will be no meetings to be held*, *this basically depends on the program*. *If so*, *then we run more errands assigned to each*. *At noon*, *we go out to evangelize and have lunch afterwards*. *We then drink our coffee and we exercise*. *We take a break*, *have a bath*, *then we either have dinner or hold a meeting right before dinner*. *We sometimes get our own free time after dinner and this depends on the schedule*. *We then say our prayers and go straight to bed*. *That’s our daily routine*.*” (0304; male 23 years)*
		*“Coming from a free world where you have access to coffee*, *soft drinks wherever you are and then all of a sudden you have a structured life*, *this is frustrating*.*” (0403; female 31 years)*
		*“The food here is way better than the food outside*. *It’s healthier*.*” (0209; male 21 years)*
		***“The available*** *foods are not so healthy*.*” (0405; female 25 years)*
		*“I was never satisfied with my weight gain*, *I cry day and night*.*” (0405; female 25 years)*
		*“Yes*, *a bit of weights*, *a bit of stretching*, *a bit of cardio*. *In summer it is very nice we play basketball*, *volleyball*, *football*. *Stuff like that*, *and we also go for other activities in summer*, *so we enjoy the weekend*.*” (0401; female 23 years)*
		*“Whoever sleeps a lot during the day is noticed by the management*. *To them*, *this person is facing issues in the treatment*, *so they speak to him privately to determine his weakness and work on it*.*” (0208; male 24 years)*
		*“I need someone to wake me up like four times in the morning*.*” (0401; female 23 years)*
		*“Do you need more sleep*?*” (Facilitator) “Yes*.*” (0401 & 0403; females 23 and 31 years)*
	*Therapy*	*“When I arrived here*, *I was in a very bad shape*, *I had no communication with others*, *and I was introverted*. *Yet*, *as soon as you arrive*, *they start working on your weaknesses and boost your self-confidence*. *They also set goals for whoever is afraid of confronting his/her fear*.*” (0209; male 21 years)*
***Suggestions for making rehabilitation a better experience***	*Nutrition*	*“I feel that what is wrong in this center is that instead of giving sweets as a snack to the person who is hungry or asking him to wait till the the next meal*, *he/she should be given an apple or a banana to stop the feeling of hunger and to stop him/her from the continuous thinking of food*.*” (0401; female 23 years)*
		*“we have rules and we do not want more to be added” (0403; female 31 years)*
	*Physical activity*	*“Would you prefer to have physical activity first thing in the morning*, *or it does not matter*?*” (facilitator)“In the afternoon*. *When I do sports*, *it’s the only time I do not have drug cravings” (0403; female 31 years)*

## Discussion

This study is among the first to briefly shed the light on the lifestyle of PUWD including eating, sleep and exercise behaviours during addiction with more emphasis on the early and late stages of rehabilitation. Participants described their disciplined lifestyle in rehabilitation centers, which were overall well-received. But, they identified pitfalls which, if not properly addressed, may lead some to relapse. They suggested measures for improving rehabilitation services.

Our participants discussed factors leading to substance use disorder and rehabilitation that are echoed in the literature [43–46]. Further, lifestyle practices during addiction including low food and poor nutrient intakes [2, 26, 47], lack of engagement in physical activity, as well as disrupted sleep due to the pronounced effect of drugs on wakefulness are also reported globally [19, 20, 22].

We found that residential rehabilitation centers provide a stable environment to prevent relapse. The focus is mainly on pharmacotherapy and psychotherapy as means for preventing relapse; and secondly on enforcing a disciplined routine to regain normality in lifestyle practices. While the former seem to be overall well-received, the latter was a blanket approach, not addressing individuals’ preferences, thus suboptimal and in need of being redressed. Empowering individuals to gain a healthy lifestyle practices during rehabilitation is important to prevent relapse as our participants indicated. In fact, binge eating and weight gain were associated with relapse, especially among females [48, 49]; and lack of sleep may be bidirectional: drug use causes sleep disturbances and difficulty sleeping causes relapse [50]. Thus, providing person-centered interventions including personal coaching in rehabilitation centers by way of preventing relapse are suggested as essential components in treatment facilities [51, 52].

In terms of eating practices, in the early stage of rehabilitation, binge eating is sometimes the sole divergent from drugs [48, 53, 54]. Effective measures for controlling food intake may include pairing nutritional programs with leisure/vocational activities, to establish healthy eating behavious while simultaneously increasing self-worth through actively working with individuals and identifying skills and vocations effaced during addiction. Further, establishing nutrition educational programs with emphasis on increasing knowledge, and changing attitudes and practices to promote positive nutrition behaviour [26, 51] could help preventing relapse [55].

Physical activity is another lifestyle behaviour poorly addressed in rehabilitation centers in Lebanon. Our participants almost marginalized the role of physical activity by labeling it as ‘boring’. It is an essential element as it reduces relapse and withdrawal sufferings while improving sleep [21, 30, 56]. It also has a positive effect on the psycho-social wellbeing of individuals [24, 56], where a growing body of literature suggests that it yields mental health and wellness benefits. Brown et al. [57] addressed this gap in the literature and developed an exercise intervention as an adjunct to addiction treatment for drug dependent patients. This study demonstrated benefits in increasing days of abstinence from drugs and alcohol. However, it was challenging to enroll participants in this study, and meet the physical demands of the program. Precise recommendations on the type, amount and frequency of physical activity remains elusive and further studies should consider the adverse effect besides the benefits [38, 58]. Interventions to incent individuals to participate in physical activities, while improving the quality and variety of programs are highly recommended. Fun Sports that encourage team work and communication tend to have an appeal for some participants and serve as a forum for the practice of social skills and the development of friendships with other recovering individuals. Understanding motivational differences among participants is a key determinant of engagement and adherence [13, 29, 59]. Thus, when identifying the type and level of physical activity, it is important to have them person-centered, i.e. taking into consideration individuals’ self-efficacy, readiness, and preference of the type of activity offered.

The benefits conferred by physical activity, proper nutrition and adequate sleep have been independently associated with better mental health and physical wellbeing in substance use disorder. These benefits occur through multicomponent effects on the neurobiological and psychosocial development. Studies evaluating strategies to enhance maintenance of treatment have devoted little attention to lifestyle modification, and information related to this area is still scarce [57]. Investigating the impact of interventions related to these lifestyle domains within clinical practices to enhance treatment and prevent relapse is highly recommended [38].

Follow-up treatment in the community beyond the rehabilitation center is crucial to decrease the risk of relapse among users, and was expressed as a need among most of our participants. There is accumulating evidence suggesting the association between the length of the treatment modality and drug use relapse. The longer the treatment (6–12 months), the less the relapse [60]. The research indicates the need to investigate further the factors that contribute to sustaining a decrease in drug use and negative behaviours post-treatment.

## Study strengths and limitations

This study pioneered in tackling the lifestyle practices of PWUD undergoing treatment in rehabilitation centers in Lebanon through qualitative research in the absence of any quantitative data. There are several strengths and limitations that are worth noting. This study fills a gap in the international literature on the lifestyle of PWUD undergoing residential rehabilitation. The research team was composed of two female members: NM and GHA who are located in Lebanon. GHA, the qualitative methodologist, worked with NM, a researcher in health promotion, on developing the predetermined open-ended questions to guide the discussion and conducted interviews. Our evaluation employed several techniques to ensure reflexivity and to increase the credibility of the study: securing a high proportion of potentially eligible individuals to participate, using transcribed audio-recorded interviews, and using participants’ quotes to support our findings. Furthermore, the attrition rate in this study was low as twenty-seven out of the twenty-nine who were invited to participate enrolled in this study and finished it. Both researchers have no prior rapport with the participants or the rehabilitation centers.

As for the limitations, female participants were less represented compared to the males and this goes back to the limited number of rehabilitation centers in Lebanon that accommodate females.

Factors associated with poor lifestyle behaviours including psychotropic medications and cigarette smoking were not adequately addressed. Furthermore, it is possible that because participants were still actively involved in the rehabilitation services when collecting the data, their responses may have been more socially desirable to avoid offending their host institution. Finally, there is a limited generalizability of the results, because of the non-random sample and the contextual restrictions where only three rehabilitation centers granted us access.

## Implications for future research

Residential treatment centers are controlled environments with potential for offering and implementing healthy lifestyle intervention programs to its residents. Policies for better treatment to PWUD talking all aspects of behavioural changes can be developed from extensive research in this population group. Further research in assessing the nutritional status and healthy behaviours of people who use drugs in rehabilitation centers are required for the development and implementation of a multidisplinary intervention program in the promotion of good health among this population group. Also it is important to test the evidence generated by this qualitative research through quantitatve ones, ultimately to come out with an evidence based multifaceted intervention.

## Conclusion

PWUD undergoing treatment in rehabilitation centers are a vulnerable population with many challenges. Treatment services mainly concentrate on the medical management of withdrawal and its complications with little emphasis on other treatment modalities. In this study, we shed the light on some of the challenges PWUD face in maintaining a healthy lifestyle in rehabilitation centers and the necessities of addressing those challenges in order to improve the overall rehabilitation experience and prevent relapse.

## Supporting information

S1 Appendix Study guide. (DOCX)

S2 Appendix Thematic inductive analytical approach. (DOCX)

S3 Appendix Consolidated criteria for reporting qualitative studies (COREQ): 32-item checklist. (DOCX)
