# Supplementary material for: People who use drugs in rehabilitation, from chaos to discipline: Advantages and pitfalls: A qualitative study
Source: PLoS One. 2021 Feb 5;16(2):e0245346. doi: 10.1371/journal.pone.0245346 (PMC7864414; doi:10.1371/journal.pone.0245346)
Supplement: S1 Appendix — (DOCX) [file pone.0245346.s001.docx]

# **S1 Appendix. Study guide.**

**Gender:**

**Age:**

**Marital Status:**

**Focus group Guide Questions**

1. Tell me a little about yourself and why you are here
2. Let us talk about your daily routine
3. What do you usually eat?
4. Who selects the food?
5. Who cooks the food?
6. Let us talk about the effect of the drug/treatment on the food intake and food choices
7. Let us talk about other factors that you feel affects your food intake
8. What do you think about your individual and group eating habits?
9. Let us talk about your experiences with weight change
10. How do you feel about your weight ?
11. What in your opinion causes the weight loss/ gain ?
12. What do you think about having information on healthy eating ?
13. What are you interested in knowing about food and nutrition?
14. What do you think is the best way to gain such information?
15. Let us talk about exercise
16. What type of exercise do you do ?
17. Would you like to do more exercise as part of the program?
18. What ideas do you have to increase exercise ?
