# Supplementary material for: People who use drugs in rehabilitation, from chaos to discipline: Advantages and pitfalls: A qualitative study
Source: PLoS One. 2021 Feb 5;16(2):e0245346. doi: 10.1371/journal.pone.0245346 (PMC7864414; doi:10.1371/journal.pone.0245346)
Supplement: S2 Appendix — (DOCX) [file pone.0245346.s002.docx]

# **S2 Appendix. Thematic inductive analytical approach.**

Adopted from: Braun V, Clarke V. Using Thematic Analysis in Psychology.Qualitative Research in Psychology.2006:77-101

Data was thematically analyzed along six phases.

**Phase 1:** The interviewers and coder read and re-read each transcript to get acquainted with the information.

**Phase 2**: An initial list of codes was generated.

**Phase 3**: The search for themes started. The interviewer and coder discussed the relationships between codes. A log of potential themes and sub-themes was developed, including a list of definitions and quotes to illustrate each theme and sub-theme.

**Phase 4:** The list of themes was further refined based on consensus reached among all research team members to define themes and sub-themes and highlight the existing relationships between these themes.

**Phase 5**: Defining and refining the themes was done, that is we identified the story behind each theme and the relationship between themes.

**Phase 6**: The findings were presented in a narrative form, and a synthesis of the results was included. These findings were supported with quotes from interviewees and beneficiaries relating to identified themes and sub-themes. In this stage, every effort was made to provide a concise, coherent, logical, non-repetitive, and interesting account of the story the data tell – within and across themes.
